# Editorial: Parasite-induced liver diseases, volume II

**DOI:** 10.3389/fcimb.2026.1940191

**Published:** 2026-08-03

**Authors:** Xiaobin Fan, Xing He

**Affiliations:** Department of Tropical Diseases, Shanghai Key Laboratory of Medical Bioprotection, Key Laboratory of Biological Defense, Ministry of Education, Naval Medical University, Shanghai, China

**Keywords:** fibrosis, inflammation, microbia, parasite infection, treatment

Parasitic infections remain a major cause of liver disease worldwide, contributing substantially to the global burden of cirrhosis, portal hypertension, and hepatocellular carcinoma (Hong et al., 2024). The liver, with its unique tolerogenic properties and continuous exposure to gut-derived antigens, provides an immunological niche that many parasites exploit to establish chronic infection. Despite advances in antiparasitic chemotherapy, the hepatic sequelae which ranging from persistent inflammation and progressive fibrosis to malignant transformation often continue unabated, largely because host inflammatory and fibrotic responses are not effectively targeted by current treatments. This Research Topic, *Parasite-Induced Liver Diseases Volume II*, was conceived to bring together studies that probe the cellular and molecular underpinnings of these pathologies and explore new avenues for diagnosis and therapy. The nine articles collected here, spanning immune regulation, therapeutic intervention, microbiome interactions, and technological innovation, offer a multidimensional view of the field.

A central theme of this Research Topic is the diversity of immune checkpoint mechanisms that parasites exploit to subvert hepatic immunity. Tang et al. mapped hepatic CD8^+^ T-cell evolution during alveolar echinococcosis (AE). They found that chronic *Echinococcus multilocularis* (*E. multilocularis*) infection drives the concurrent expansion of cytotoxic, effector-memory, and exhausted CD8^+^ T-cell subsets, with coordinated upregulation of negative regulatory genes (*Btg1*, *Tnfaip3*, *Junb*, *Nr4a1*) and downregulation of early inflammatory mediators. These findings align with a growing body of evidence showing that T-cell exhaustion is a hallmark of chronic AE (Yang et al., 2024). Indeed, recent work has demonstrated that depletion of myeloid-derived suppressor cells can restore T-cell function and enhance antiparasitic immunity in AE (Zhang et al., 2024a). Furthermore, Wang et al. showed that Tim-4^+^ macrophages, which accumulate in AE lesions, suppress proinflammatory cytokine production and promote immune tolerance. Importantly, inhibition of Tim-4 not only restored inflammatory balance but also reversed hepatic fibrotic progression *in vitro*, pointing to a dual role for this molecule in immune evasion and tissue remodeling. In the context of schistosomiasis, Zhu et al. uncovered a novel regulatory axis in which IL-10, acting through STAT5, suppresses miR-140 to upregulate the immune checkpoint B7-H4 in macrophages. B7-H4, a co-inhibitory molecule of the B7 family, has been implicated in the suppression of T-cell responses in various cancers and chronic infections. Collectively, these studies illustrate how phylogenetically distant parasites—cestodes and trematodes—converge on overlapping immunoregulatory pathways to establish persistent infection in the liver.

The challenge of therapeutic intervention is addressed by two studies evaluating natural products with anti-fibrotic and antiparasitic properties. Luo et al. demonstrated that total flavonoids of litchi seed (TFL), a traditional Chinese medicine rich in rutin, quercetin, and morin, attenuated *Clonorchis sinensis* (*C. sinensis*)-induced liver fibrosis in rats. TFL improved liver function, reduced hepatic stellate cell (HSC) activation, and downregulated collagen I/III and α-SMA expression, both *in vivo* and in TGF-β1-stimulated HSC-T6 cells. These effects are consistent with recent reports showing that *C. sinensis* excretory-secretory products promote fibrosis through intercellular crosstalk between cholangiocytes and HSCs, and that gut microbiota-derived metabolites may modulate this process (Yi et al., 2024). Notably, TFL exerted its anti-fibrotic effects without directly killing the parasite, suggesting a host-directed mechanism. Similarly, Zhu et al. found that oxymatrine, an alkaloid from Sophora flavescens Aiton, directly inhibited *E. multilocularis* protoscolex viability *in vitro* and reduced cyst burdens *in vivo*. Mechanistically, oxymatrine increased hepatic CD8^+^ T-cell abundance and enhanced gut microbial diversity. Oxymatrine has previously been shown to inhibit HSC activation and reduce expression of profibrotic cytokines; the present study extends this knowledge by demonstrating its dual action on parasite viability and host immune-microbiome modulation. Together, these two studies highlight the therapeutic potential of natural products through mechanisms that engage both the parasite and the host response.

The gut microbiome and virome as modulators of host-parasite interactions are explored in two studies. Shang et al. reconstructed >6,000 wild rodent gut genomes, revealing that *Enterocytozoon bieneusi* infection alters bile acid-metabolizing taxa. The absence of 7β-HSDH in laboratory mice highlights limitations of conventional models and supports wild rodents as more relevant for bile acid research, particularly given evidence that enteric infections remodel the bile metabolome (Zhang et al., 2024b). Muribaculaceae, especially CAG-485, emerged as a key bile salt hydrolase (BSH)-encoding lineage, with BSH-positive genomes enriched in glycoside hydrolases, suggesting a link between bile acid and carbohydrate metabolism. Expanding this analysis to the viral component, Yu et al. identified 178 differentially abundant viral operational taxonomic units (161 enriched in infected groups), with infection altering virome diversity and virus-bacteria correlations; functional analysis showed enrichment of pathways related to terpenoid, polyketide, cofactor, and vitamin metabolism. These findings reinforce the gut-liver axis concept, wherein intestinal microbial and viral communities influence hepatic inflammation and fibrosis via metabolic and immunological signals (Tripathi et al., 2018).

Finally, this Research Topic embraces technological innovation to uncover previously inaccessible layers of complexity. Ma et al. applied imaging mass cytometry (IMC) to human AE liver tissues, resolving 16 immune cell types with spatial resolution. They identified distinct spatial architectures, such as CD15^+^ neutrophils in partially necrotic zones, M1/M2 macrophages and dendritic cells forming ring-like structures around CTL-rich areas, and observed heterogeneous, cell type-dependent expression of immune checkpoints. IMC has recently emerged as a powerful tool for mapping the immune landscape of liver diseases; its application to parasitic liver disease in this study represents a significant advance. On the epigenetic frontier, Wei et al. provided the first comprehensive genome-wide maps of 5-methylcytosine and 5-hydroxymethylcytosine in *C. sinensis*-infected hepatocellular carcinoma at single-nucleotide resolution. They identified 29 differentially methylated regions and 13 differentially hydroxymethylated regions between infected and non-infected tumors, associated with 28 genes, two of which (*DHDH* and *KCNQ3*) correlated with overall survival. These findings are consistent with a growing recognition that parasitic infections can reshape host DNA methylation patterns and chromatin accessibility, highlighting the importance of epigenetic remodeling in parasite-associated hepatocarcinogenesis (Kafle et al., 2024; Yang et al., 2025).

The articles collected in this volume reveal the multifaceted strategies by which parasites subvert hepatic immunity and drive pathology, while also pointing to new therapeutic and diagnostic opportunities. We trust this Research Topic will foster cross-disciplinary dialogue and accelerate the translation of mechanistic discoveries into improved outcomes for patients with parasite-induced liver diseases.
